# Revisiting Asthma Obstructive Sleep Apnea Overlap: Current Knowledge and Future Needs

**DOI:** 10.3390/jcm12206552

**Published:** 2023-10-16

**Authors:** Damini Saxena, Ikuyo Imayama, Muhammad Adrish

**Affiliations:** 1 Section of Pulmonary, Critical Care and Sleep Medicine, Baylor College of Medicine, Houston, TX 77030, USA; 2 Division of Pulmonary, Critical Care, Sleep and Allergy, University of Illinois, Chicago, IL 60607, USA

**Keywords:** asthma, obstructive sleep apnea, overlap

## Abstract

Asthma and obstructive sleep apnea are highly prevalent conditions with a high cost burden. In addition to shared risk factors, existing data suggest a bidirectional relationship between asthma and OSA, where each condition can impact the other. Patients with asthma often complain of sleep fragmentation, nocturnal asthma symptoms, daytime sleepiness, and snoring. The prevalence of OSA increases with asthma severity, as evidenced by multiple large studies. Asthma may lower the threshold for arousal in OSA, resulting in the hypopnea with arousal phenotype. Epidemiologic studies in adults have shown that OSA is associated with worse asthma severity, increased frequency of exacerbation, and poor quality of life. The current literature assessing the relationship among OSA, asthma, and CPAP therapy is heavily dependent on observational studies. There is a need for randomized controlled trials to minimize the interference of confounding shared risk factors.

## 1. Introduction

The Centers for Disease Control and Prevention (CDC) estimates that 21 million adults suffer from asthma in the U.S. [1]. According to a 2013 estimate, asthma cost more than 80 billion USD in the U.S. [2]. It is estimated that the total direct costs of asthma will be 1537 billion USD from 2019 to 2038; however, if all patients achieve asthma control during the next 20 years, 300.6 billion USD (20%) in direct costs can be saved [3]. There is an urgent need to improve asthma care. As awareness of undiagnosed obstructive sleep apnea (OSA) grew in the late 20th century, its diagnosis saw a sharp rise. The number of patients with OSA needing office visits increased from 2.0 million in 2000 to 2.7 million in 2010 [4]. In 2015, the estimated cost of diagnosing and treating OSA in the U.S. was around 12.4 billion USD, while the cost of undiagnosed OSA was a staggering 149.6 billion USD [4]. This highlights the urgent need for improved strategies in diagnosing and caring for these patients.

The reciprocal relationship between asthma and obstructive sleep apnea (OSA) is well-documented in population surveys and meta-analyses [5,6,7,8]. Patients with asthma often complain of sleep fragmentation, nocturnal asthma symptoms, daytime sleepiness, and snoring [9,10]. A 2-3-times-higher prevalence of OSA has been reported in patients with asthma [9,11,12]. The large-scale European Sleep Apnea Database (ESADA) study found that only 5% of patients with OSA were diagnosed with comorbid asthma, suggesting that asthma patients are underdiagnosed with concurrent OSA, or, alternatively, OSA patients are inadequately screened for underlying asthma [13]. Notably, the prevalence of OSA in asthma may vary on depending on the type of sleep studies used for its diagnosis. Polysomnographic-diagnosed OSA is prevalent in patients with uncontrolled asthma, but OSA diagnosed by respiratory polygraphy (sleep test without an electroencephalogram) is less common in the same patients [11]. Respiratory polygraphy, which is becoming ubiquitous in diagnosis, may lead to an underdiagnosis of OSA in asthma by missing respiratory events with arousal [14]. Underdiagnosis is amplified by the under-recognition of OSA in asthma.

Existing data suggest a bidirectional relationship between asthma and OSA, where each condition can impact the other. Asthma is recognized as a risk factor for OSA and can worsen its severity [15]. Conversely, OSA has been associated with asthma development and its effects on the condition’s progression [11]. In this review, we will examine recent clinical findings to provide a comprehensive overview of the interplay between asthma and OSA and suggest potential avenues for future research in this area.

## 2. Effects of Asthma on OSA

The prevalence of OSA increases with asthma severity, as evidenced by multiple large studies [9,16]. The severe asthma group had higher prevalence of OSA compared with the moderate asthma group (88% vs. 58%) [13]. Symptoms of uncontrolled daytime and nighttime asthma were more prevalent in those with OSA compared with those with no OSA [15]. Furthermore, Teodorescu and colleagues reported a higher incidence of OSA in asthmatics (relative risk 2.72) and that a longer asthma duration (5-year increment) was associated with an increased risk of OSA (relative risk 1.07) using data from the Wisconsin Sleep Cohort study [14,15].

Though OSA prevalence increases with asthma severity, the severity of OSA does not correlate with asthma diagnosis, nor asthma severity [9,14]. A large survey found a similar distribution of OSA severity (48% were assessed by polysomnogram) in patients with and without asthma [12]. A subsequent metanalysis also showed that asthma had no significant effect on OSA; however, it is noteworthy that most of the patients in this analysis were from a single study, which may have driven its results [6,13]. The Sleep Heart Health Study noted that the severity of OSA assessed by polysomnography in non-asthma patients was higher than that in asthma patients [5]. This discrepancy was attributed to variation between cohorts, as the non-asthma control was predominantly male and older—known risk factors for severe OSA—while the asthma cohort was predominantly female and younger. A low arousal threshold may prompt arousal or awakening, resulting in fewer respiratory events in asthma. Further studies on this are needed.

### 2.1. How Asthma Affects OSA Phenotype

Asthma may lower the threshold for arousal in OSA, resulting in the hypopnea with arousal phenotype [11]. A study by Bellia and colleagues evaluated the relationship between nocturnal asthma and sleep [17]. Catheters were placed in the esophagus and supraglottic area to measure upper and lower airway resistance. The authors noted an increase in lower airway resistance and bronchoconstriction episodes during stages 3 and 4 of sleep. These findings were further studied by Ballard and colleagues, who compared lower airway resistance in six subjects with asthma and four control subjects on three separate occasions (i.e., night of normal sleep, one night awake, and one night after sleep deprivation) [18]. Patients with asthma had higher airway resistance during the night of normal sleep and sleep prevention night [18]. A low arousal threshold (AT) is considered one of the four pathophysiological traits of OSA. A retrospective study analyzed whether low AT was a common pathophysiological factor in OSA and asthma by retrospectively analyzing the polysomnographic records of thirty-five patients with asthma and OSA and thirty-six patients with OSA alone. The study noted that those with OSA and asthma were more than twice as likely to have low AT compared with patients with OSA and without asthma [19].

### 2.2. Pathological Explanations of How Asthma Affects OSA

#### 2.2.1. Direct Mechanical Effects

Asthma patients are at higher risk of nasal obstruction related to nasal congestion and chronic inflammation (Table 1). The reduced caliber of nasal passages increases upper airway collapse [6,9,20]. Upper airway narrowing in asthma patients may be present without comorbid allergic rhinitis and nasal polyposis. Acoustic rhinometry and peak nasal inspiratory flows have measured smaller nasal airway cross-sectional areas in asthma patients with and without allergic rhinitis [21]. Nasal obstruction results in intranasal resistance, which increases negative pressures in the upper airway during inspiration and contributes to the pathogenesis of OSA [20].

#### 2.2.2. Lung Volumes and Airway Resistance

Hyperinflation, increased functional residual capacity (FRC), is one characteristic of asthma. Increased FRC allows the trachea to exert a pulling effect on pharyngeal tissues, supplementing upper airway patency [9,22]. Ballard et al. observed that FRC decreased during sleep for both asthmatics and non-asthmatics, while the degree of FRC reduction was greater in asthmatics [23]. A reduction in FRC during sleep can precipitate loss of airway patency and collapse [9], increasing the risk of OSA events in asthma [8,19]. Tamisier and colleagues analyzed pharyngeal pressure-to-flow ratios to evaluate upper airway resistance (UAR) variations during inspiration and expiration in eleven patients with moderate–severe sleep-disordered breathing. While both inspiratory and expiratory resistance increased during sleep, the increase in UAR during expiration occurred earlier than that during inspiration and was significantly aggravated during the last three breaths immediately preceding total upper airway collapse during an obstructive event [24]. These findings suggest that the premature closure of the upper airway during sleep may not only impact the expiratory airflow but may also lead to significant stress on the lower airway structures. Mechanical stimulation of the airway has been linked to the development of early proinflammatory processes in the upper airway [25].

#### 2.2.3. Bronchoconstriction

A correlation between bronchoconstriction and sleep fragmentation has been seen, though it is unclear in which phase of sleep the effects of bronchoconstriction are the worst [26]. One study found that the forced expiratory volume in 1 s (FEV1) and peak expiratory flow (PEF) dropped significantly during REM sleep and may have been a result of severe bronchoconstriction in nocturnal asthma [27]. Other studies similarly observed that asthmatic wheezing and oxygen desaturation were the worst during REM sleep [28,29]. However, Belia et al. measured lower airway resistance to be higher in asthma during non-REM sleep [17]. Meanwhile, other studies have found no correlation between bronchoconstriction and sleep stage [9]. The variability in findings may be due to variability in the methodologies of assessing bronchoconstriction.

#### 2.2.4. Neuroinflammatory Mechanisms

The pharyngeal upper airway is maintained patent by several opposing muscles that depend on complex neural pathways. To understand the impact of non-adrenergic non-cholinergic (NANC) system overnight airway caliber, Mackay and colleagues compared the bronchodilator effects of capsaicin (an NANC stimulant) on 12 normal and 12 subjects with asthma at 04:00 h and 16:00 h. The authors noted a greater bronchodilation at 16:00 h compared with 04:00 h suggesting that NANC functioning may contribute to overnight bronchospasm [30]. This may be explained by the elevated levels of eosinophils and macrophages in the alveolar tissue at 04:00 in the morning [26]. Increased airway inflammation has been associated with loss of N3 sleep, but not REM sleep [31,32]. Sleep fragmentation, together with poor sleep quality, may lead to ventilatory instability.

#### 2.2.5. Type 1 Inflammation

**Figure 1:**
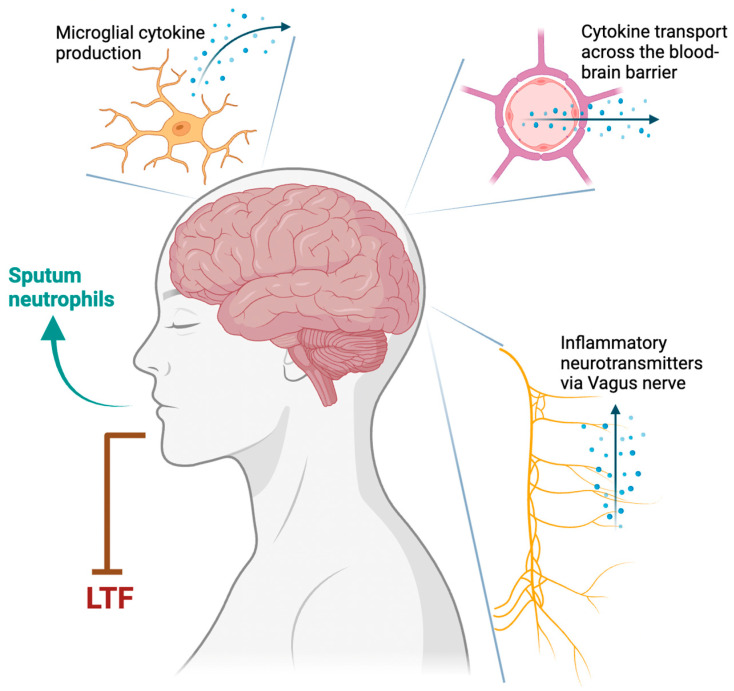
Proposed mechanisms of Type 1 inflammation in asthma destabilizing respiratory control in obstructive sleep apnea. LTF: long-term facilitation.

Type 1 inflammation is usually characterized by the presence of airway neutrophilia and absence of airway eosinophilia. Various cytokines, including interleukin (IL)-6, IL-8, IL-17, IL-1β, tumor necrosis factor (TNF), and interferon-gamma, may play a role in its pathogenesis [33]. Using bleomycin-induced lung injury in rats, Jacono et al. proposed three pathways linking T1 inflammation to respiratory control: the transport of cytokines across the blood-brain barrier, transport of inflammatory neurotransmitters through the Vagus nerve, and cytokine production by microglia (Figure 1) [34]. These pathways are heightened by peripheral chemoreceptors sensitized to T1 inflammation. Chronic inflammation in asthma attenuates the force generated by respiratory muscles, resulting in upper airway collapse [35]. Animal studies have shown that lung inflammation destabilizes breath control, which may contribute to OSA [11].

Neutrophilic inflammation in T1 asthma induces OSA [26]. The Severe Asthma Research Program (SARP II) study found that asthma patients with high risk of OSA had 1.4 times increase in neutrophils compared with asthma patients with a low risk of OSA [15]. Another study showed elevated sputum neutrophils in patients with asthma and OSA compared with asthma without OSA [36]. This may be due to the elevated neutrophil-derived metalloproteinase-9 and IL-8, resulting in basement membrane thinning, which correlated with a higher AHI [36]. A study by Vicente and colleagues compared pharyngeal lavage and plasma samples for IL-6 and TNF levels among patients with OSA, healthy controls, and snorers [37]. The authors noted higher levels of these inflammatory biomarkers in the pharyngeal lavage, but not in the plasma.

Long-term facilitation is a form of airway plasticity that causes augmented neural activity [11]. T1 inflammation may contribute to upper airway patency via LTF. Neural pathways can activate upper airway muscles for prolonged periods, called LTF. Animal studies have shown that the duration of LTF is subject to inflammation [11]. Lipopolysaccharide-mediated T1 inflammation reduces LTF and thus upper airway patency, while anti-inflammatory substances will reverse these effects. This phenomenon has been visualized in a chronic allergic rat model, in which chronic intermittent hypoxia resulted in T1 inflammation and blunted LTF, predisposing to OSA [38]. This same response was seen in utero in newborn animal studies [11]. Chronic intermittent hypoxia in pregnancy and postpartum resulted in suppressed LTF and increased respiratory instability in newborns, promoting OSA development later in life.

#### 2.2.6. Type 2 Inflammation

Type 2 (T2) airway responses are primarily mediated by eosinophils, basophils, and mast cells [39,40]. T2 helper T cells and T2 innate lymphoid cells, along with cytokines, including IL-4, IL-5, and IL13, play a role in its pathogenesis. Broytman and colleagues conducted their study on rats, in which rats receiving ovalbumin, which induces an IgE-mediated response, were compared with normal saline [41]. Ventilatory oxygen and carbon dioxide equivalents were measured under room air and hypoxic conditions. The ovalbumin rats demonstrated hyperventilation in response to hypoxia, suggesting that IgE-mediated T2 inflammation destabilizes respiration and may lead to hyperventilation-induced apnea during sleep. L-arginine metabolism is dysregulated in T2 asthma, which may facilitate OSA. In one study, L-arginine metabolites in patients’ sera were measured and correlated with their OSA status, spirometry, asthma control, and morbidity [42]. Approximately 54% of patients in this study had T2 inflammation and about 81% of the study participants were on inhaled corticosteroids (ICS). Patients with asthma and concurrent OSA had higher levels of the nitric oxide synthase inhibitor asymmetric dimethylarginine, lower L-arginine levels, and higher ornithine and proline levels compared with asthma patients without OSA, suggesting shared pathophysiology between T2 asthma and OSA.

Fractional exhaled nitric oxide (FeNO) is a biomarker of type 2 airway inflammation [40]. It has been shown to correlate with lung function and future exacerbations and predict the response to treatment in patients with asthma [40]. Tichanon and colleagues studied airway inflammation in patients with OSA and assessed the effect of continuous positive airway pressure (CPAP) therapy. Patients with OSA had higher FeNO levels at baseline compared with normal controls, which reduced significantly after CPAP therapy. Similar findings were noted in other studies implicating the role of upper airway inflammation in OSA pathogenesis [43].

### 2.3. How Asthma Treatments Affect OSA

ICS may increase the risk of OSA (Table 2) [44]. Canine models previously showed that corticosteroids antagonize smooth muscle bronchoconstriction in the trachea via the p38 MAPK pathway [45]. This process has paradoxical effects on pharyngeal muscles, causing muscle fiber atrophy and central neck fat redistribution, promoting OSA [9]. In one survey, ICS was correlated with an increased risk of developing OSA dose-dependently, irrespective of asthma severity (odds ratio = 4.1) [46]. High-dose fluticasone over 4 months in asthma increased tongue strength during wakefulness and decreased endurance, as seen in OSA [47]. The short duration of this study suggests that a substantial population on lifelong ICS may have pharyngeal myopathy and fat redistribution, elevating their risk of OSA. Long-term ICS use also modulates the release of growth hormone, inducing metabolic and cardiovascular complications that worsen the effects of OSA in comorbid individuals [10]. These findings are noteworthy given that current GINA guidelines encourage the use of ICS, both as a controller and reliever of asthma [48]. ICS particle size affects the development of OSA in asthma [49]. Patients inhaling extra-fine particles of ICS, defined as smaller than 2 µm, had a similar risk of developing OSA compared with controls not using ICS. Inhalers containing standard-sized particles increased the risk of OSA. Large ICS particles will preferentially deposit in the upper airway and posterior pharynx, causing local adverse effects, compared with finer particles that reach terminal lung bronchioles. This finding may impact which ICS inhaler is used in asthma patients prone to OSA. A prospective cohort study examining continuous or “burst” dose oral corticosteroids in uncontrolled asthma found that 95% of patients had concomitant OSA, thought to be [36] related to adverse effects of systemic corticosteroid use, including upper airway remodeling and worsening obesity [50].

In contrast, intranasal corticosteroids in patients with allergic rhinosinusitis have improved the severity of OSA. Targeting the inflammation of nasal passages reduces airflow resistance and improves upper airway patency [51]. Santos and colleagues conducted a study to evaluate the effect of montelukast on sleep disturbances in patients with perennial allergic rhinitis. In this double-blind placebo-controlled study, patients treated with montelukast showed a significant improvement in daytime fatigue and somnolence [52]. These findings suggest that the treatment of rhinitis may have beneficial effects in patients with OSA.

With the advent of biological treatments that preferentially target T2 inflammation in asthma, potential therapeutic targets have emerged that need further investigation [53]. A recent case series of five patients with severe asthma with chronic rhinosinusitis, nasal polyposis, and OSA found a remarkable reduction in OSA symptoms in all patients after 6 months of therapy with dupilumab, an IL-4 and IL-13 antagonist [54]. Similarly, in another case, AHI halved after 6 months of asthma therapy with the anti-IgE antibody omalizumab [55].

### 2.4. Effect of OSA on Asthma

Asthma–OSA overlap syndrome, the presence of both OSA and asthma, is associated with worse morbidity and mortality compared with either disease alone [56]. A cohort study of 4980 veterans showed a 10-year all-cause mortality of 63.5% for asthma–OSA overlap, 54.2% for asthma alone, and 60.4% for OSA alone [57]. In patients with OSA not receiving treatment (positive airway pressure (PAP) therapy), the risk of death was 1.34 (1.05–1.71) times higher than that of those on PAP therapy [57]. In patients with OSA nonadherent to PAP therapy, the risk of death was 1.78 (1.13–2.82) times higher compared with that of those who were compliant to PAP therapy (≥70% of nights and >4 h/night) [57]. Therefore, OSA could be a modifiable target to improve the clinical outcomes of people with asthma.

### 2.5. OSA Affects Asthma Severity and Control

Epidemiological studies in adults have shown that OSA is associated with worse asthma severity, increased frequency of exacerbation, and poor quality of life (Table 3). Cross-sectional studies have shown that high OSA risk and OSA in asthma are associated with more severe asthma, poor asthma control, and impaired quality of life. In 137 asthmatic adults, people who were at low OSA risk according to the Berlin questionnaire were more likely to have controlled asthma than those with a high OSA risk (odds ratio 7.896, 95% CI 2.902–21.487) [58]. Among 472 subjects with asthma, high OSA risk was associated with 2.87 increased odds of having not-well-controlled asthma [59]. In 217 adults with asthma, the high-OSA-risk group had a lower score in asthma-specific quality of life compared with the low-OSA-risk group, while the asthma control test (ACT) scores were not statistically different [60]. In 813 asthma subjects, OSA was associated with 6.67 times increased risk of severe asthma in older subjects (age 60–75) and 2.16 times increased risk in younger subjects (age 18–59) [61,62]. In 136 patients with difficult-to-treat asthma, OSA was associated with 3.4 times (95% CI 1.2–10.4) increased risk of frequent asthma exacerbation [62]. In 90 subjects attending a difficult asthma clinic, the presence of OSA was associated with worse ACT scores and asthma-related quality of life, which became not statistically significant after accounting for body weight [63].

The associations between OSA and poor asthma control and exacerbation have also been examined in longitudinal studies. In the 10–11 years follow-up of the World Trade Center Health Registry (*n* = 2445), there was a trend of higher OSA prevalence with worse asthma control (40.6% in very poorly controlled, 34.2% in poorly controlled, and 17.6% in controlled) [64]. OSA was associated with 1.39 times increased risk of poorly controlled asthma, and 1.48 increased risk of very poorly controlled asthma, adjusting for covariates including body mass index (BMI) [64]. In a prospective study of 146 asthmatics and 157 matched controls, OSA was associated with higher risk of severe asthma exacerbations (relative risk 14.23, 95% CI 4.60–44.04 vs. no OSA) [65]. AHI was significantly associated with the occurrence of severe asthma exacerbations (odds ratio 1.322, 95% CI 1.148–1.523) [65]. In a 5-year follow-up of 177 subjects with difficult-to-control asthma, OSA was not associated with an increased risk of frequent severe exacerbation [66].

PAP therapy to treat OSA has been shown to improve asthma outcomes, especially in severe OSA or poorly controlled asthma (Table 4) [67]. In a survey of 1586 subjects who were on CPAP therapy for OSA, asthma was present in 13% [68]. Among 152 subjects who started CPAP after starting asthma therapy, CPAP use was 6.3 h per day [68]. The self-reported asthma severity decreased significantly from 48.3 (29.6) to 33.1 (27.4), and ACT score increased significantly from 15.35 (5.3) to 19.8 (4.6) without a significant change in BMI [68]. The percentage of patients using rescue medication daily decreased from 36% to 8% with CPAP [68]. Six-week CPAP therapy improved AHI and quality of life, but not airway responsiveness, as measured by the provocation test in 20 adults with asthma and newly diagnosed OSA [69]. In 100 people with asthma referred for sleep evaluation, 54% had severe, 33% had moderate, and 13% had mild OSA [70]. After 3 months of CPAP, clinical asthma control was good in 70% (vs. 41% at baseline) and the ACT score increased to 21 ± 4 (vs. 19 ± 4 at baseline) [70]. In a prospective study of 99 adults with asthma and OSA, 6 months of CPAP improved asthma control, with increased Asthma Control Questionnaire score (1.39 ± 0.91 at baseline to 1.0 ± 0.78 at 6 months), decreased percentage of patients with uncontrolled asthma from 41.4% to 17.2% and percentage of patients with asthma attacks in the 6 months before and after treatment from 35.4% to 17.2%, and increased asthma-specific quality of life [71]. In 37 subjects with asthma and OSA (AHI ≥ 10/hour) randomized to 3-month CPAP and control groups, no differences in ACT score were seen between the two groups, but the CPAP group had lower daytime sleepiness and better quality of life scores [72].

As described above, current evidence on associations among OSA, asthma, and PAP therapy is mainly derived from observational studies. Intervention studies, especially randomized controlled trials (RCTs), are needed to understand the impact of PAP therapy and optimal PAP usage to achieve better asthma control. In addition, we were unable to identify studies that investigated the use of alternative therapy for OSA, such as hypoglossal nerve stimulator, dental appliance, and mandibular advancement surgery, on asthma outcomes.

### 2.6. How OSA Affects Pulmonary Function in Asthmatic Subjects

Studies have shown that OSA may worsen pulmonary function in asthmatics; however, its reversibility with OSA treatment is controversial. Body weight could partially explain a decline in pulmonary function with OSA diagnosis. A recent meta-analysis of nine studies showed that the percentage of predicted FEV1 (%FEV1) tended to decrease in adult asthma patients complicated with OSA, but the trend did not reach statistical significance [6]. In a case–control study of 30 asthmatics vs. 12 age-, gender-, and BMI-matched controls, the subjects with asthma and OSA had higher BMI (29.2 ± 3.3 vs. 25.4 ± 4.4) and a lower FEV1 value (72.8 ± 4.8 vs. 78.2 ± 2.6) compared with asthmatics without OSA [75]. High BMI, GERD, and %FEV1 were independent predictors of OSA in this study. In a retrospective study of 466 subjects recruited through a sleep laboratory, there was a dose-dependent association between a decrease in FEV1 and increase in AHI [76]. The annual decline in FEV1 was greater in those with severe OSA (72.4 ± 61.7 mL) compared with those with mild to moderate OSA (5 ≤ AHI < 30 per hour, 41.9 ± 45.3 mL) and the normal group (AHI < 5 per hour, 24.3 ± 27.3 mL); meanwhile, BMI was not associated with an annual decline in FEV1 [76]. The decline in FEV1 decreased from 69.4 ± 66.4 mL to 41.2 ± 36.1 mL after CPAP therapy [76]. In a cohort study of 4329 subjects who attended at baseline and a 10-year follow-up, FEV1 and FVC declined more rapidly in subjects with high OSA risk compared with those with low OSA risk (FEV1 –50.8 ± 30.1 mL/year vs. –41.3 ± 24.3 mL/year; FVC −45.2 ± 36.3 mL/year vs. −30.5 ± 31.2 mL/year) [77]. These declines were associated with increased BMI between visits and OSA symptoms [77]. When the analysis was adjusted for changes in weight, only those with asthma had a significant association between the OSA symptom score and decline in lung function, suggesting that OSA may impact asthma outcomes independently of weight changes [77].

There is conflicting evidence regarding whether PAP therapy attenuates airway responsiveness. A 6-week CPAP therapy improved AHI, but not airway responsiveness, as measured by a provocation test in 20 adults with asthma and newly diagnosed OSA [69]. A prospective study of 99 asthmatic adults with OSA reported a reduced percentage of subjects with positive bronchodilator response after 6 months of CPAP therapy (36% to 12%) [71]. In 57 people with OSA and 13 controls, exhaled NO remained higher in OSA subjects compared with the controls, even after using CPAP therapy [78]. Airway responsiveness to methacholine did not differ between OSA and controls at the baseline, but airway responsiveness increased after CPAP use [78]. In 16 asthmatic adults, nasal CPAP use was associated with an enhanced bronchodilator response to salbutamol and reduced airway responsiveness to methacholine [79]. Another study in stable asthma with normal spirometry showed reduced airway reactivity to methacholine with nocturnal CPAP use for 7 days [80].

Very limited evidence exists on the effects of alternative OSA therapy on asthma. In a survey of subjects with asthma and OSA on oral appliances, 1-month of oral appliance therapy improved AHI and asthma control [81]. The lack of objective measurements of adherence and comparison with CPAP requires further investigation.

### 2.7. Pathological Explanations of How OSA Affects Asthma

Numerous studies have reported multiple pathological mechanisms to explain how OSA affects asthma control. In the following sections, we will review the current literature on the following mechanisms, with a specific focus on studies examining the effects of OSA treatment: obesity, hypoxemia, frequent arousals, increased negative intrathoracic pressure, autonomic nerve system, and airway inflammation.

#### 2.7.1. Obesity

Obesity is an established risk factor for both OSA and asthma. Approximately 60% of moderate-to-severe OSA cases are attributed to excess body weight [82]. Using data from a cohort study, Peppard and colleagues estimated that 10% weight gain increased AHI by 32%, while 10% weight loss decreased AHI by 26% [83]. Similarly, weight gain has been shown to increase the risk of asthma in adults [84]. Using data from National Health and Nutrition Examination Surgery (NHANES), people gaining > 29 kg from young to middle adulthood had a 1.53 times increased risk of asthma (95% CI 1.15 to 2.03) compared with those with stable weight (weight change ≤ 2.5 kg) [84]. Weight loss interventions using dietary and/or exercise, as well as bariatric surgery, have been shown to improve asthma control [85,86,87]. A meta-analysis of six randomized controlled trials reporting successful weight loss in all studies showed improved asthma-related quality of life, asthma control, and lung function (FEV_1_, FVC, and total lung capacity) [88].

Some epidemiological studies have shown that the significant associations between OSA and asthma are attenuated after accounting for parameters of obesity, suggesting obesity as a partial mediator of the association between OSA and asthma [63,75,89]. To our knowledge, we are unaware of any weight loss trials specifically targeting people with asthma and OSA overlap syndromes. Future trials would benefit from exploring the impacts of weight control in people with overlap syndrome, and the necessary degree of weight loss to achieve a clinically significant improvement in asthma control to guide optimal weight for those with overlap syndrome.

#### 2.7.2. Hypoxemia

Animal models have shown that hypoxia may enhance bronchial responsiveness. In sheep, bronchial responsiveness to methacholine was increased by hypoxia and the response was mediated by carotid body chemoreceptors [90]. Teodorescu and colleagues showed that chronic intermittent hypoxia increased airway hyperresponsiveness to methacholine challenge in rats exposed to house dust mites and intermittent hypoxia [91]. Intermittent hypoxia for 6 h/d for 14 days increased methacholine-induced airway hyperresponsiveness and inflammatory marks in bronchoalveolar fluid in rats [92]. The daily use of N-acetylcysteine or ibuprofen completely nullified these responses [92]. In contrast, Sultonov and colleagues reported a decreased response to allergen response in mice treated with intermittent hypoxia compared with those without exposure to intermittent hypoxia [93].

People with asthma and OSA have lower nocturnal oxygen saturation than those with asthma or OSA only. Participants with both wheezing and OSA had a significantly lower nocturnal oxygen saturation (92.5 ± 0.5%) than participants with wheezing only (94.3 ± 0.3%) and OSA only (93.6 ± 0.2%) [94]. In 384 women, the group with both asthma and OSA had lower mean oxygen saturation (93.4% vs. 94.7%) than the group with OSA alone [95].

Although OSA may be associated with lower oxygen saturation, the impact of hypoxemia on asthma control is yet to be established. In a randomized cross-over study of 12 healthy male subjects, including 2 with a history of asthma, Saito and colleagues showed no differences in airway responsiveness to methacholine between hypoxic and normoxic days [96]. In 16 OSA and 10 control subjects, bronchial wall thickness and blood inflammatory biomarker levels were greater in OSA vs. controls [97]. Bronchial wall thickness was positively associated with AHI, BMI, respiratory arousal index, and duration of SPO_2_ < 90%, but AHI was the only predictor of bronchial wall thickness in the adjusted model [97]. To our knowledge, no studies have examined how changes in oxygen saturation after the initiation of PAP therapy alter bronchial responsiveness or asthma control. Future studies are needed in this area.

#### 2.7.3. Frequent Arousals and Poor Sleep Quality

Symptoms of insomnia are often seen in OSA. These symptoms may mediate the associations between OSA and asthma. Insomnia symptoms, difficulties in falling asleep and maintaining sleep, are highly prevalent in asthma, especially in those with uncontrolled asthma [98]. In a cross-sectional study of 200 asthmatics visiting an outpatient clinic, the severity of insomnia was inversely related to the level of asthma control: moderate-to-severe insomnia was more frequent in patients with uncontrolled asthma (43%) than in those with partially controlled asthma (25%) or well-controlled asthma (12%) [99]. Uncontrolled asthma remained significantly associated with insomnia symptoms, difficulty in initiating sleep (OR 1.72 (1.15–2.56)), difficulty in maintaining sleep (OR 1.55 1.09–2.21), and early morning arousals (OR 1.77 1.12–2.82) after adjusting for age, sex, educational level, BMI, smoking history, comorbidities, and physical activity [86,98]. In 714 adults with asthma enrolled in SARP III, those with insomnia were at 2.4-times increased risk of having not-well-controlled asthma and 1.5-times increased risk of asthma-related health care utilization in the past year compared with those without insomnia [100]. A cross-sectional analysis of 1150 asthmatics showed that uncontrolled asthma (ACT score < 20) was associated with 3.3 times higher odds of having poor sleep quality (Pittsburgh Sleep Quality Index score > 5) [101].

In 384 women, those who had both asthma and OSA had more frequent awakenings, longer time in N1 and N2 sleep, and less time in REM sleep than the control group [95]. Whether the observed insomnia symptoms are independently associated with asthma control or symptoms, or they are mediated through other comorbid sleep disorders, including OSA, is yet to be determined. There is a randomized controlled trial testing the impact of cognitive behavioral therapy for insomnia on sleep symptoms and asthma control in chronic asthma and insomnia [102]. The findings of this trial may provide insights into how insomnia symptoms may affect asthma control.

#### 2.7.4. Negative Intrathoracic Pressure

Negative intrathoracic pressure, a hallmark of OSA, increases both right ventricular pre-load and left ventricular afterload, and can trigger pulmonary congestion [103]. In addition, hypoxemia from OSA can trigger cardiac ischemia, further worsening pulmonary congestion [104]. Asthmatics with nocturnal worsening have increased pulmonary capillary volume and decreased single-breath alveolar volume, FEV1, and FEV1/FVC ratio, while normal subjects and asthmatics without nocturnal worsening do not exhibit any changes in these parameters [105]. Untreated OSA may worsen nocturnal asthma symptoms through triggering pulmonary congestion.

#### 2.7.5. Autonomic Nervous System

OSA modulates the autonomic nervous system through multiple mechanisms [106]. The partial or complete collapse of the upper airway during apneic episodes increases vagal tone, triggering bronchoconstriction and nocturnal asthma symptoms [20]. Mechanical irritation of the laryngeal mucosa triggers a reflex that increases the total lung resistance distal to the larynx and parasympathetic activity of the trachea and bronchi [107]. In a case–control study of 26 patients with OSA and 19 age- and sex-matched controls without OSA, parasympathetic activity was increased during the night in subjects with OSA, while no differences in parasympathetic activity were seen after 2-month CPAP therapy between the OSA and control groups [108]. However, in a systematic review mostly investigating cardiovascular outcomes in OSA, the overall characteristics of the autonomic nervous system in OSA were increased sympathetic activity and reduced parasympathetic activity [109]. Treating OSA resulted in decreases in muscle sympathetic nerve activity and catecholamine levels [110]. This contrasts with the hypothesis that increased vagal tones trigger asthma symptoms in OSA. Although the precise mechanism is yet to be established, the local trigger of the vagal nerve system from mechanical irrigation may differ from that of the cardiac autonomic system. In addition, homeostasis to correct the imbalance of the sympathetic and parasympathetic systems may contribute to increased nocturnal vagal tone, triggering asthma symptoms in OSA.

#### 2.7.6. Airway Inflammation

OSA may affect asthma through increasing airway inflammation. In a study of asthma and normal subjects, higher OSA risk was associated with increased % sputum neutrophils, which suggests that OSA may be an important contributor to neutrophilic asthma [111]. In 35 patients with newly diagnosed moderate to severe OSA, exhaled breath condensates (nitrotyrosine, IL-6, tumor necrosis factor (TNF)-alpha, and 8-isoprostane) and some serum inflammatory biomarkers (nitrotyrosine and 8-isoprostane) decreased after 3-month CPAP therapy (59). Exhaled breath condensates of nitrotyrosine were associated with a higher oxygen desaturation index and duration of SpO_2_ < 90% [112]. In a meta-analysis, OSA increased fractional exhaled nitric oxide (FeNO) in patients with asthma (pooled weight mean difference of 4.37, 95% CI 0.05–8.69) (5) [6]. In OSA, proximal airway resistance was positively associated with sputum levels of inflammation (IL-8 and TNF-alpha) accounting for BMI [113]. The sputum levels of inflammation (IL-8 and TNF-alpha) were related to a duration of SpO_2_ < 90% [113].

Leptin is a hormone produced in adipose tissue that regulates energy homeostasis, inflammation, metabolism, and sympathetic nervous system activity. An in vivo study has shown that leptin increases airway hyperresponsiveness [114]. However, the serum level of leptin has not been consistently associated with OSA or weight gain. There were no changes in the serum leptin levels in OSA treated with auto-adjusting CPAP for 6 months [115]. Leptin resistance, an inability to respond to leptin, is a mechanism that may explain why a high serum leptin level may not reflect elevated leptin functioning in humans [116].

## 3. Conclusions

Numerous studies have revealed a bi-directional relationship and proposed the underlying pathophysiology of explaining interactions between OSA and asthma. The current literature investigating the associations between asthma, OSA, and CPAP therapy is largely based on observational studies. A randomized controlled trial to assess the impacts of CPAP therapy on asthma severity and control is needed to further evaluate the association. This is particularly important, as asthma and OSA share risk factors such as obesity, which could confound the associations.

## 4. Future Directions

Asthma is a heterogeneous disease with multiple disease mechanisms [33,117]. Variable classifications are used, such as type 2, neutrophilic, or mixed inflammation and phenotypes (clinical presentations) [33,118]. Future studies may benefit from focusing on the impacts of OSA and/or CPAP therapy within each subgroup. Although AHI or a parameter of hypoxemia (nadir SpO_2_ or duration of SpO_2_ < 90%) has been used as a measure of OSA severity, the optimal parameter to assess the impact of OSA on asthma is yet to be determined. In addition, not all studies examined parameters to assure sufficient treatment of OSA with CPAP therapy. Improving the assessment of OSA severity and efficiency of CPAP therapy is needed.

The current literature assessing the relationship among OSA, asthma, and CPAP therapy is heavily dependent on observational studies. OSA and asthma share multiple overlapping factors, such as obesity, rhinitis, and GERD. Therefore, there is a need for randomized controlled trials to minimize the interference of these confounding factors.

## Figures and Tables

**Table 1:** Hypothesis of how the severity of asthma increases the prevalence of OSA, including proposed mechanisms via specific pathological pathways.

Proposed Mechanisms	Pathological Pathway
∆ Sleep architecture	↓ FRC ↓ Long-term facilitation Respiratory phase interdependence
↑ Nasal congestion	
↑ Type 1 inflammation	↑ Bronchoconstriction ↑ Sputum neutrophils
↑ Type 2 inflammation	↑ NANC inhibition

OSA: obstructive sleep apnea, FRC: functional residual capacity, NANC: non-adrenergic non-cholinergic neural pathways, ↑: increased, ↓: decreased, ∆: change in.

**Table 2:** Effect of asthma treatments on OSA.

Treatment Type	Risk of Developing OSA	Mechanisms
Inhaled corticosteroids (ICS)	↑	↑ Central neck fat distribution, pharyngeal muscle myopathy
ICS extra-fine particles (<2 µm)	↓	↓ Upper airway deposition of particles
Oral corticosteroids	↑	↑ Obesity, ↑ central neck fat distribution, pharyngeal muscle myopathy
Intranasal corticosteroids	↓	↓ Airway resistance, ↑ airway patency
Antileukotrienes	↓	↓ Airway resistance, ↑ airway patency
Biologic therapy	↓	Unclear

↑: increased, ↓: decreased.

**Table 3:** Impact of OSA on asthma severity and control.

Author	Year	Sample	Definition of OSA	Findings
*Cross sectional*				
ten Brinke [62]	2005	*n* = 136 difficult-to-treat asthma	PSG or history of snoring and daytime sleepiness with frequent apnea periods of >10 s	↑ frequent exacerbation OR 3.4 (1.2–10.4) adjusted for age and asthma duration. ↔ frequent exacerbation after accounting for covariates
Teodorescu [59]	2010	*n* = 472 outpatient clinic	Sleep Apnea Scale of the Sleep Disorders Questionnaire (high OSA risk vs. without high risk)	↑ uncontrolled asthma in high OSA risk. OR 2.87 (95% CI 1.54–5.32) accounting for covariates
Kim [60]	2013	*n* = 217 outpatient clinic	Berlin questionnaire (high risk vs. low risk)	↓ asthma specific quality of life score in high OSA risk (vs. low OSA risk) ↔ asthma control
Teodorescu [61]	2013	*n* = 813 outpatient clinic	PSG	↑ worse asthma severity step (OR 2.91, 95% CI 1.15–7.36) ↑ severe asthma (OR 6.67, 95% CI 1.74–25.56) in older subjects (age 60–75) vs. OR 2.61, 95% CI 1.28–5.33) in younger subjects (age 18–59).
Tay [63]	2016	*n* = 90 difficult asthma clinic	Berlin questionnaire (high OSA risk vs. low risk)	↔ frequent exacerbation, ACT score, and asthma-specific quality of life accounting for increasing BMI and other comorbid conditions
Ozden Mat [58]	2021	*n* = 137 outpatient clinic	Berlin questionnaire (high OSA risk vs. low risk)	↑ 7.9 times increased odds of uncontrolled asthma Odds ratio 7.896, 95% CI 2.902–21.487.
*Longitudinal*				
Jordan [64]	2015	*n* = 2445 World Trade Center Health Registry, 10–11 years follow up	Physician-diagnosed OSA	↑ 1.39 and 1.48 times increased risk of poorly controlled asthma and very poorly controlled asthma, respectively, adjusting for covariates
Wang [65]	2016	*n* = 146 asthma *n* = 157 no asthma 1 year follow up	PSG	↑ AHI increased risk of severe asthma exacerbations (OR 1.322, 95% CI 1.148–1.523)
Yii [66]	2017	*n* = 177 Step 4 of GINA treatment ladder 5 years follow up	PSG	↔ severe asthma exacerbation

AHI, apnea hypopnea index; BMI, body mass index; CI, confidence interval; OR, odds ratio; OSA, obstructive sleep apnea; PSG, polysomnography; ↑: increased, ↓: decreased, ↔: unchanged.

**Table 4:** Impact of CPAP therapy on asthma severity and control.

Author	Year	Sample	Study Design	Findings
Lafond [69]	2007	*n* = 20 OSA and asthma	Prospective, 6 weeks after CPAP therapy	CPAP use 6.7 h/d ↔ airway responsiveness, %FEV1, FEV1/FVC ratio ↑ asthma-specific quality of life
Teodorescu [16]	2012	*n* = 75 CPAP therapy, OSA and asthma	Cross-sectional	↓ persistent daytime asthma symptoms ↔ persistent nighttime asthma symptoms
Shaarawy [73]	2013	*n* = 15 uncontrolled asthma and OSA	Prospective, 6 weeks after CPAP therapy	↓ Epworth sleepiness scale ↓ arousal index ↔ % FEV1, %FVC, FEV1/FVC ratio ↔ ACT score
Kauppi [68]	2016	*n* = 152 CPAP started after asthma treatment	Cross-sectional, survey questionnaire	CPAP use 6.3 h/d, mean 5.7 years. ↓ self-reported asthma severity and ↑ACT score without significant changes in BMI ↓ daily rescue medication use
Serrano-Pariente [71]	2017	*n* = 99 OSA and asthma	Prospective, before and after 6 months of CPAP	↓ asthma control questionnaire score ↓ % of uncontrolled asthma ↓ % of asthma attacks ↓ GERD symptoms ↓ positive bronchodilation test ↓ FeNO ↑ Asthma control and ↑ quality of life among patients compliant with CPAP (≥4 h/night) vs. noncompliant subjects.
Shaker [74]	2017	*n* = 12 OSA and asthma	Prospective, 3 months after CPAP therapy	↓ daytime and nighttime asthma symptoms ↓ GERD symptoms ↓ difficult to control asthma ↓ Epworth sleepiness scale ↑ %FEV1 ↑ FEV1/FVC ratio ↑ sleep efficiency ↓ total sleep time
Ng [72]	2018	*n* = 17 CPAP group *n* = 20 control group Nocturnal asthma symptoms and OSA	Randomized controlled trial	CPAP use, 5.0 h/d at 1 month and 5.2 h/d at 3 months ↓ Epworth sleepiness score ↑ asthma specific quality of life ↑ vital domain of quality of life ↔ ACT score ↔ asthma exacerbation rate, spirometry, and airway responsiveness
Cisneros [70]	2023	*n* = 100 OSA and asthma	Retrospective, before and ≥3 months after CPAP	↑ clinical asthma control ↑ ACT score

ACT, asthma control test; CPAP, continuous positive airway pressure; FeNO, fractional exhaled nitric oxide; FEV1, forced expiratory volume in 1 s; FVC, forced vital capacity; GERD, gastroesophageal reflux disease; OSA, obstructive sleep apnea; PSG, polysomnography; ↑: increased, ↓: decreased, ↔: unchanged.

## Data Availability

Not applicable.
